# Readthrough of Premature Termination Codons in the Adenomatous Polyposis Coli Gene Restores Its Biological Activity in Human Cancer Cells

**DOI:** 10.1371/journal.pone.0024125

**Published:** 2011-08-31

**Authors:** Célia Floquet, Jean-Pierre Rousset, Laure Bidou

**Affiliations:** 1 Institut de Génétique et Microbiologie, CNRS, UMR 8621, Orsay, France; 2 Université Paris-Sud, Orsay, France; 3 Université Pierre et Marie Curie, Paris, France; Virginia Commonwealth University, United States of America

## Abstract

The APC tumor suppressor gene is frequently mutated in human colorectal cancer, with nonsense mutations accounting for 30% of all mutations in this gene. Reintroduction of the WT APC gene into cancer cells generally reduces tumorigenicity or induces apoptosis. In this study, we explored the possibility of using drugs to induce premature termination codon (PTC) readthrough (aminoglycosides, negamycin), as a means of reactivating endogenous APC. By quantifying the readthrough of 11 nonsense mutations in APC, we were able to identify those giving the highest levels of readthrough after treatment. For these mutations, we demonstrated that aminoglycoside or negamycin treatment led to a recovery of the biological activity of APC in cancer cell lines, and showed that the level of APC activity was proportional to the level of induced readthrough. These findings show that treatment with readthrough inducers should be considered as a potential strategy for treating cancers caused by nonsense mutations APC gene. They also provide a rational basis for identifying mutations responsive to readthrough inducers.

## Introduction

The APC (adenomatous polyposis coli) tumor suppressor gene encodes a 311 kDa multidomain protein with binding sites for numerous partners, including microtubules, axin and beta-catenin [1]. This protein functions as a negative regulator of the Wnt pathway which is implicated in the transactivation of target genes such as the oncogene c-myc and the cyclin D1 gene [2]. The APC gene is mutated in 80% of colon cancers and mutations occur early in the development of most polyploid colorectal cancers. It has been shown that reintroduction of the wild-type APC gene into cancer cells generally reduces tumorigenicity or induces apoptosis [3], [4]. Overall, 30% of all mutations in this gene are nonsense mutations [5].

In the last few years, certain antibiotics have been shown to interfere with the mammalian ribosome, inducing the readthrough of premature stop codons (PTCs). Aminoglycosides are the antibiotics most frequently studied in this respect, and multiple studies have suggested that these drugs could be used in innovative strategies for the treatment of genetic diseases caused by nonsense mutations (for review [6], [7], [8]). The dipeptide antibiotic negamycin is not structurally related to aminoglycosides [9] and has been found to be more effective than gentamicin for promoting the readthrough of some nonsense mutation [10]. This drug may thus constitute an attractive alternative treatment for patients carrying a nonsense mutation.

In a recent study, we demonstrated that even a modest aminoglycoside-induced readthrough level (6%) triggered apoptosis of cancer cells harboring a nonsense-mutated p53 gene [11]. These results provided the proof-of-concept that readthrough inducers can be used in the treatment of cancers linked to the presence of a nonsense mutation in a tumor suppressor gene. In the present study, we investigate the possibility of extending this approach to cancer linked to a nonsense mutation in APC gene. Recently, Zilberberg et al showed that readthrough inducers improved clinical symptoms of tumorigenesis in a mouse model carrying a nonsense mutation in the APC gene. However, the link between the lower level of tumor development and PTC readthrough was not established [12]. It is becoming increasingly evident that only a subset of nonsense mutations would benefit from gentamicin treatment, depending on their nucleotide context [13], [14], [15], [16].

We evaluated readthrough efficiency for 11 nonsense mutations involved in colorectal cancer, with a view to identifying the nonsense mutations in the APC gene the most responsive to readthrough-inducing treatments. For the mutations with the highest readthrough levels, including an endogenous mutation, antibiotic treatment resulted in recovery of the biological activity of APC.

## Results and Discussion

### Identification of APC nonsense mutations responsive to aminoglycoside treatment

**Figure 1:**
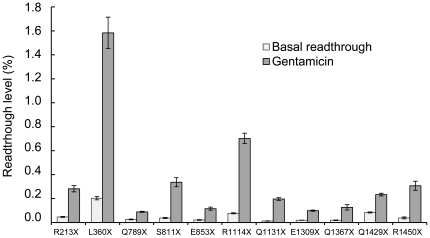
Identification of APC nonsense mutations responsive to aminoglycoside treatment. Readthrough efficiencies for 11 nonsense mutations in the APC gene were assessed in NIH3T3 cells with and without gentamicin (800 µg/ml) treatment for 24 h. Two nonsense mutations (L360X and R1114X) displayed levels of gentamicin-induced readthrough of more than 0.5%. Means values are presented, together with the standard error of the mean (SEM) (n = 5).

We studied 11 nonsense mutations found in 23% of cancer cells carrying a nonsense mutation in the APC gene (T. Soussi, personal communication). Readthrough efficiency has been shown to depend on the nature of the sequences surrounding the stop codon [13], [17], [18], [19]. Thus, for each mutation, we inserted a region encompassing the surrounding context (27 nucleotides) into the dual reporter vector pAC99 (Table 1). Readthrough was quantified in NIH3T3 cells transiently transfected with the dual reporter vector, in the presence or absence of gentamicin (Figure 1). Readthrough rates ranged from 0.01% (Q1131X) to 0.2% (L360X) for basal readthrough, and from 0.09% (APC Q789X) to 1.6% (L360X) in the presence of 800 µg/ml gentamicin. Similar variations in basal readthrough levels and responsiveness to aminoglycosides have been reported in several previous studies [13], [18]. We also tested another antibiotic, amikacin, which gave readthrough levels similar to or lower than those obtained with gentamicin (data not shown). This result is consistent with a previous study from Howard et al who have compared readthrough activity of several aminoglycosides [20].

**Table 1:** APC nonsense mutations.

WT Codon	Mutation *	Sequence 5′>3′§	Mutation Frequency †
CGA	R213X	GAT ATG GAA AAA **TGA** GCA CAG CGA	2.6%
TTA	L360X	CTC ATC CAG CTT **TGA** CAT GGC AAT	‡
CAG	Q789X	CAT CGT AGT AAG **TAG** AGA CAC AAG	0.1%
TCA	S811X	GAT GAT AAT AGG **TGA** GAC AAT TTT	0.3%
GAG	E853X	GAT AGA AGT TTG **TAG** AGA GAA CGC	0.3%
CGA	R1114X	TCA GAA ACA AAT **TGA** GTG GGT TCT	2.6%
CAA	Q1131X	CAG TCT TTG TGT **TAA** GAA GAT GAC	0.1%
GAA	E1309X	GCA GAA ATA AAA **TAA** AAG ATT GGA	1.8%
CAG	Q1367X	AAA AGT GGT GCT **TAG** ACA CCC AAA	1.8%
CAA	Q1429X	GAT AGC CCT GGA **TAA** ACC ATG CCA	0.9%
CGA	R1450X	GCT CAA ACC AAG **TGA** GAA GTA CCT	12.5%
All mutations studied			23.2%

*Mutations are named by the position and the nature of the wild-type amino acid in APC protein sequence.

§ These nonsense mutation sequences were inserted into the dual reporter vector in order to determine readthrough level.

† Frequencies were given relative to total nonsense mutations listed for APC gene.

‡ This nonsense mutation was reported in [33].

We assessed whether readthrough levels were similar in cells of colon origin, by also evaluating the readthrough directed by each nonsense mutation in the cancer colorectal DLD-1 cell line. The results obtained were similar to those for NIH3T3 cells (data not shown).

**Figure 2:**
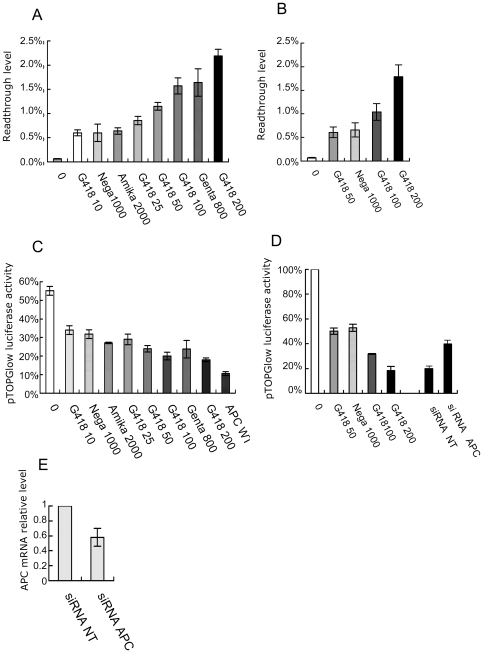
APC biological activity is correlated with antibiotic-induced readthrough level in human colorectal cancer cells. (A) Readthrough efficiencies for APC L360X nonsense mutations were determined in DLD-1 cells in the presence of G418 (10, 25, 50, 100 and 200 µg/ml), negamycin (1 mg/ml), amikacin (2 mg/ml) or gentamicin (800 µg/ml). (B) Readthrough efficiencies for APC R1114X nonsense mutations were determined in NIH3T3 cells in the presence of G418 (50, 100 and 200 µg/ml) or negamycin (1 mg/ml). The readthrough efficiencies in DLD-1 cells were consistent with those in NIH3T3 cells (data not shown). (C) Aminoglycoside treatment restored APC activity. APC binding to beta-catenin (reppression of the pTOPGlow reporter) is restored by the treatment of DLD-1 cells transiently transfected with cDNA APC L360X with G418 (10, 25, 50, 100 and 200 µg/ml), negamycin (1 mg/ml), amikacin (2 mg/ml) or gentamicin (800 µg/ml). (D) APC binding to beta-catenin (repression of pTOPGlow reporter) is restored by the treatment of LoVo cells carrying APC R1114X with G418 (50, 100 and 200 µg/ml) or negamycin (1 mg/ml). As a control, LoVo cells were cotransfected with the pTOPGlow reporter plasmid and either the APC-targeting siRNA (siRNA APC) or a non-targeting siRNA (siRNA NT) and treated with G418 (200 µg/ml). (E) Effect of the siRNA targeting APC mRNA. We transiently transfected LoVo cells with an siRNA targeting (siRNA APC) or not targeting (siRNA NT) APC mRNA. Quantitative PCR was used to determine mRNA levels. Results are expressed relative to the amount of mRNA in the presence of siRNA NT. Mean values are presented, together with the SEM (n = 3).

For further characterization, we focused on the L360X and R1114X nonsense mutations, which displayed the highest levels of readthrough in the presence of the aminoglycoside gentamicin (1.6% and 0.7%, respectively). For the two selected nonsense mutations, we quantified the readthrough levels obtained in the presence of various concentrations of G418 (geneticin), because this antibiotic is the most potent readthrough inducer of the aminoglycosides [11], [21], [22]. Both nonsense mutations displayed a dose-dependent response to G418, resulting in readthrough levels greater than those observed for gentamicin, reaching 2.3% for L360X and 1.8% for R1114X (Figure 2 A and 2B respectively). The readthrough levels obtained in the presence of the dipeptide negamycin were lower than (L360X) or similar (R1114X) to those obtained with gentamicin (Figure 2 A and 2B respectively).

Readthrough level depends on the nature of the stop codon and the surrounding nucleotide context, but the rules governing readthrough level are complex and remain to be determined for mammalian cells. The presence of a C residue immediately after the stop codon (+4) is correlated with a high level of readthrough, although this does not seem to be the sole determinant of readthrough level. Several studies have also demonstrated that only a few nonsense mutations give “significant” readthrough in the presence of gentamicin. In this study, only two of the eleven nonsense mutations tested, L360X and R1114X, displayed gentamicin-induced readthrough levels greater than 0.5%, but only L360X had a C residue in the +4 position. The R1114X mutation would therefore have been discarded if this had been the only criterion used to identify mutations responding well to readthrough inducers. Our approach thus provides a rational strategy for identifying patients likely to benefit from treatment with readthrough inducers.

### Recovery of APC biological activity from the APC L360X cDNA after aminoglycoside treatment

We then investigated whether aminoglycoside treatment induced detectable levels of biological activity for an APC protein produced from a cDNA bearing the L360X mutation. Active APC mediates the sequestration of beta-catenin in the cytoplasm, thus preventing the interaction of this molecule with the transactivation factor TCF required for the expression of target genes. APC protein activity was evaluated by determining the ability of this protein to bind beta-catenin, using a reporter construct containing TCF/beta-catenin binding sites inserted upstream from the luciferase firefly gene (pTopGlow) [23]. In this reporter system, the interaction between active APC and beta-catenin results in the inhibition of firefly luciferase expression.

DLD-1 cells devoid of functional APC protein (APC del 1 bp 1406) were transfected with the pCMV expression vector containing either WT or L360X mutant APC cDNA, together with pTopGlow (Figure 2C). Transfection with the WT APC vector resulted in levels of firefly expression one tenth those obtained after transfection with an empty vector. Without treatment, L360X displayed residual levels of activity, probably due to the incomplete loss-of-function of this allele, as residual activity has also been demonstrated for mutations in nearby positions [23]. The highest dose of G418 decreased luciferase activity by a factor of 2.5, demonstrating the production of a functional APC protein on aminoglycoside treatment. G418 repressed luciferase activity in a dose-dependent manner correlated with the readthrough levels measured in DLD-1 cells (Figure 2A). Treatment with the aminoglycosides gentamicin or amikacin, or with negamycin also triggered the repression of luciferase activity, indicating the synthesis of an active APC protein (Figure 2C). For each treatment, protein activity was correlated with the level of readthrough level measured in the dual reporter system (Figure 2A and 2C). We used Spearman's nonparametric correlation test (two-tailed) to assess the significance of this correlation. We found that there was a strong, highly significant correlation (rs  = −0.95, p<0.0001) between readthrough level and the decrease in luciferase activity. Thus, both aminoglycoside and the dipeptide negamycin can induce APC biological activity from a mutant cDNA, and the level of activity is proportional to readthrough level. As a control, we used the pFopGlow vector containing mutated TCF/beta-catenin binding sites. With this vector, no significant change was observed after aminoglycoside or negamycin treatment (data not shown).

### Recovery of APC biological activity from the endogenous R1114X APC gene after aminoglycoside treatment

We then investigated whether the aminoglycoside treatment of endogenous nonsense mutations would give similar results. LoVo cells (APC R1114X) were transfected with the reporter plasmid pTopGlow and left untreated or treated with G418 or negamycin. The activity of the luciferase firefly reporter gene was maximal in the absence of treatment and was set at 100%, reflecting the lack of activity of the APC protein. Treatment with negamycin and G418 decreased pTopGlow firefly luciferase activity to 50% and 18% the reference value, respectively (Figure 2D). Activity level was correlated with readthrough efficiency (Figure 2B). Surprisingly, gentamicin treatment, despite promoting levels of readthrough similar to those obtained with negamycin, did not decrease pTopGlow firefly luciferase activity (data not shown). Thus, readthrough efficiency is not the only determinant for the recovery of full-length protein activity. Indeed, PTC readthrough corresponds to the incorporation of a near-cognate aminoacyl tRNA [24], [25], [26], [27] (complementary to two of the three nucleotides of a stop codon), potentially resulting in the replacement of the normal residue by an amino acid incompatible with the stability or activity of the full-length protein [10]. It is possible that different amino acids are incorporated in place of the stop codon in the presence of these two drugs, with only negamycin treatment leading to the incorporation of (an) amino acid(s) compatible with the biological activity of the protein. This situation might arise due to the different modes of activity of these drugs: aminoglycosides seem to bind exclusively to the decoding center [28], whereas negamycin has been shown to bind not only to the A site of the small ribosomal subunit [9], but also to the wall of the nascent chain exit tunnel of the large ribosomal subunit [29]. We might therefore expect the subset of natural suppressor tRNAs selected to be different after aminoglycoside and negamycin treatments.

As for the other mutation tested, luciferase expression from pFopGlow harboring mutated TCF/beta-catenin binding sites was not significantly affected by antibiotic treatment (data not shown). We checked that active APC protein was actually responsible for the observed decrease in firefly luciferase expression, using a siRNA specifically targeting the APC mRNA. We first used RT-PCR to check that the siRNA effectively decreased APC mRNA levels (figure 2E). LoVo cells were cotransfected with the pTOPGlow reporter plasmid and siRNA targeting or not targeting APC.

After G418 treatment (200 µg/ml), the APC-targeting siRNA gave a decrease in luciferase activity two times lower than that observed in absence of siRNA, whereas the non specific siRNA had no effect on luciferase inhibition (Figure 2D). Thus, the effect on the reporter system was detected specifically in the presence of APC mRNA indicating that it is mediated by an active APC protein.

**Figure 3:**
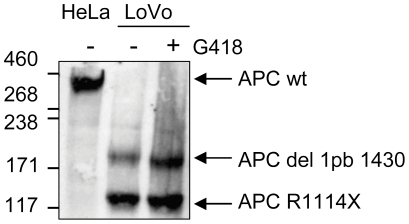
Western blot. LoVo cells were left untreated or were treated with G418 (200 µg/ml) for 72 hours. Western blots were probed with the FE-9 antibody directed against the N-terminus of APC. The truncated forms corresponding to the mutated alleles present in LoVo cells are indicated by arrows. An extract from HeLa cells (APC WT) was used as a control; a band was detected at 311 kDa.

For these two mutants, we investigated the ability of aminoglycoside treatment to restore the production of full-length APC protein. We were able to detect the full-length APC protein in HeLa cells (APC WT), but this protein was not detectable after drug treatment in either LoVo cells (R1114X) or DLD-1 cells transfected with the L360X cDNA (Figure 3). This lack of detection was probably due to the limitations of the technique for detecting small amounts of a large (311 kDa) protein. These findings suggest that low levels of APC protein may be sufficient to regulate the activity of the TCF/beta-catenin transcriptional complex.

### Conclusion

In this study, we investigated the ability of readthrough-inducing molecules to induce the production of a functional APC protein in cells carrying various nonsense mutations in the APC gene. Biological activity was detected for the two nonsense mutations most responsive to aminoglycoside treatment (R1114X and L360X) and this activity was directly proportional to the readthrough levels measured with a dual reporter system. This study shows that readthrough inducers, such as aminoglycoside and negamycin, can reactivate the APC tumor suppressor gene in cancer cells. It provides a rational strategy for identifying patients likely to respond and therefore more likely to benefit from treatment with readthrough inducers. A recent study has provided the proof-of-principle that the re-expression of a full-length APC protein after treatment with readthrough inducers reduces tumor size in a xenograft mouse [12]. Moreover, we have recently shown that the treatment of cells carrying a nonsense mutation in the p53 tumor suppressor gene leads to the re-expression of a full-length protein able to trigger apoptosis in tumor cells in culture [11]. All these data suggest that molecules inducing stop-codon readthrough could potentially be used not only to treat genetic diseases, but also to diversify the existing arsenal of treatments for cancer and as a rational basis for the development of new personalized treatment strategies.

## Materials and Methods

### Cell lines and cell culture

All cells were cultured in DMEM plus GlutaMAX (Invitrogen), supplemented with 10% fetal calf serum (FCS, Invitrogen) and 100 U/ml penicillin/streptomycin. Cells were incubated in a humidified atmosphere containing 5.5% CO_2_, at 37°C. NIH3T3 (kindly provided by Marc Sitbon) cells are embryonic mouse fibroblasts. LoVo (APC R1114X and del 1 bp 1430, kindly provided by Françoise Praz, [30]) and DLD-1 (APC del 1 bp 1406, kindly provided by Françoise Praz, [31]) cells are epithelial cells derived from a human colorectal adenocarcinoma. HeLa (APC WT) cells are epithelial cells derived from a human cervix adenocarcinoma (kindly provided by Fabrice Lejeune).

### Readthrough quantification in cell culture

Complementary oligonucleotides corresponding to nonsense mutations embedded in their natural context (sequences in Table 1) were annealed and ligated into the pAC99 dual reporter plasmid, as previously described [32]. This dual reporter allows the quantification of stop-codon readthrough, through the measurement of luciferase and beta-galactosidase (internal calibration) activities, as previously described [16]. The readthrough levels of nonsense mutations were analyzed in the presence or absence of gentamicin. NIH3T3 cells were electroporated with 20 µg of reporter plasmid and, the following day, cells were rinsed and fresh medium, with or without gentamicin, amikacin, negamycin or G418 supplementation, was added. In these experiments, no cell toxicity was observed for the doses of antibiotics used. Twenty-four hours later, cells were harvested and lysed with trypsin-EDTA (Invitrogen). Beta-galactosidase and luciferase activities were assayed as previously described [32]. Readthrough efficiency was estimated by calculating the ratio of luciferase to beta-galactosidase activity obtained with the test construct and normalizing it with respect to the ratio obtained with an in-frame control construct. For each construct, at least five independent transfection experiments were performed. For readthrough quantification in DLD-1, the same protocol was used, except that the calcium phosphate method was used for transfection. For each construct, at least three independent transfection experiments were performed.

### Expression plasmid constructs

pCMV APC WT was kindly provided by Dr. Bert Vogelstein (Johns Hopkins Oncology Center, Baltimore). Directed mutagenesis was performed on a fragment of the APC gene to create the APC L360X nonsense mutation (pCMV APC L360X). The sequence of the re-inserted fragment was verified.

### Western-blot analysis

LoVo cells were treated with G418 (200 µg/ml) for 72 h. The medium was replaced and fresh antibiotics were added each day. Cells were treated as previously described [11]. Total protein levels were determined with the Bradford reagent (Biorad) and extracts were denatured by incubation in Laemmli buffer for 5 min at 90°C. We subjected 100 µg of total protein to SDS-PAGE in NuPAGE Novex 3/8% Tris/Acetate pre-cast gels (Invitrogen). Proteins were transferred onto nitrocellulose membranes overnight, as recommended by the manufacturer. Membranes were saturated by incubation for 1 h in TBS supplemented with 5% non fat milk powder, and incubated with the primary monoclonal antibody, FE-9 (N-terminal epitope mapping between amino acid residues 1 and 35 of APC; Calbiochem, 1/100). Membranes were washed three times in TBS supplemented with 1% non fat milk powder and incubated with the secondary antibody (horseradish peroxidase-conjugated anti-mouse IgG (1/2500) or alkaline phosphatase-conjugated anti-mouse IgG (1/7000) Promega) for 45 minutes. They were washed six times and chemiluminescence was detected with ECL western blotting detection reagents (Amersham, GE Healthcare).

### Protein activity assays

The biological activity of APC was estimated with the pTOPGlow vector (kindly provided by Dr. Marc Van de Wetering, University Hospital Utrecht, the Netherlands).

DLD-1 cells were cotransfected with pCMV L360X (4 µg), pTOPGlow or pFOPGlow (10 µg) and pCMVLacZ (2 µg), by the calcium phosphate method. Each DNA precipitate was split between two wells of a six-well plate. Antibiotics (10 µg/ml to 200 µg/ml G418; 800 µg/ml gentamicin; 2 mg/ml amikacin; 1 mg/ml negamycin) were added immediately after transfection, except for G418, which was added the day after. The medium was replaced daily and fresh antibiotics were added. We prepared protein extracts 48 hours after transfection, and measured enzymatic activities.

For LoVo cells, antibiotics (G418 at concentrations of 50, 100 and 200 µg/ml; 1 mg/ml negamycin) were added the day before transfection and on each subsequent day. LoVo cells were cotransfected, by the calcium phosphate method, with TOPGlow or FOPGlow (10 µg) and pCMVLacZ (2.8 µg) to normalize for transfection efficiency, cell viability and protein extraction variability. Each DNA precipitate was split between two wells of a six-well plate. Three days after transfection, protein extracts were prepared and enzymatic activities were measured.

For each cell line, at least three independent transfection experiments were performed.

### siRNA transfections

In LoVo cells, protein activity was determined as described above. Cells were transfected with siRNA targeting mRNA APC (Dharmacon ON-TARGET plus J-003869-12) or NS, nontargeting (Dharmacon ON-TARGET plus control siRNA#1), in the presence of DharmaFECT Duo (Thermo Scientific; 1.3 µg of siRNA per well of a six-well plate), 24 h after their initial transfection as described above. Transfected cells were immediately incubated in medium with or without G418 (200 µg/ml) supplementation. Three days after transfection, the cells were harvested and luciferase and beta-galactosidase activities were assayed as described above. At least four independent transfection experiments were performed.

## References

[1] Aoki K, Taketo MM (2007). Adenomatous polyposis coli (APC): a multi-functional tumor suppressor gene.. J Cell Sci.

[2] Kolligs FT, Bommer G, Goke B (2002). Wnt/beta-catenin/tcf signaling: a critical pathway in gastrointestinal tumorigenesis.. Digestion.

[3] Groden J, Joslyn G, Samowitz W, Jones D, Bhattacharyya N (1995). Response of colon cancer cell lines to the introduction of APC, a colon-specific tumor suppressor gene.. Cancer Res.

[4] Morin PJ, Vogelstein B, Kinzler KW (1996). Apoptosis and APC in colorectal tumorigenesis.. Proc Natl Acad Sci U S A.

[5] Laurent-Puig P, Beroud C, Soussi T (1998). APC gene: database of germline and somatic mutations in human tumors and cell lines.. Nucleic Acids Res.

[6] Zingman LV, Park S, Olson TM, Alekseev AE, Terzic A (2007). Aminoglycoside-induced translational read-through in disease: overcoming nonsense mutations by pharmacogenetic therapy.. Clin Pharmacol Ther.

[7] Linde L, Kerem B (2008). Introducing sense into nonsense in treatments of human genetic diseases.. Trends Genet.

[8] Rowe SM, Clancy JP (2009). Pharmaceuticals targeting nonsense mutations in genetic diseases: progress in development.. BioDrugs.

[9] Arakawa M, Shiozuka M, Nakayama Y, Hara T, Hamada M (2003). Negamycin restores dystrophin expression in skeletal and cardiac muscles of mdx mice.. J Biochem.

[10] Allamand V, Bidou L, Arakawa M, Floquet C, Shiozuka M (2008). Drug-induced readthrough of premature stop codons leads to the stabilization of laminin alpha2 chain mRNA in CMD myotubes.. J Gene Med.

[11] Floquet C, Deforges J, Rousset JP, Bidou L (2010). Rescue of non-sense mutated p53 tumor suppressor gene by aminoglycosides.. Nucleic Acids Res.

[12] Zilberberg A, Lahav L, Rosin-Arbesfeld R (2010). Restoration of APC gene function in colorectal cancer cells by aminoglycoside- and macrolide-induced read-through of premature termination codons.. Gut.

[13] Bidou L, Hatin I, Perez N, Allamand V, Panthier JJ (2004). Premature stop codons involved in muscular dystrophies show a broad spectrum of readthrough efficiencies in response to gentamicin treatment.. Gene Ther.

[14] Howard MT, Shirts BH, Petros LM, Flanigan KM, Gesteland RF (2000). Sequence specificity of aminoglycoside-induced stop condon readthrough: potential implications for treatment of Duchenne muscular dystrophy.. Ann Neurol.

[15] Aurino S, Nigro V (2006). Readthrough strategies for stop codons in Duchenne muscular dystrophy.. Acta Myol.

[16] Sermet-Gaudelus I, Renouil M, Fajac A, Bidou L, Parbaille B (2007). In vitro prediction of stop-codon suppression by intravenous gentamicin in patients with cystic fibrosis: a pilot study.. BMC Med.

[17] Martin R, Phillips-Jones MK, Watson FJ, Hill LS (1993). Codon context effects on nonsense suppression in human cells.. Biochem Soc Trans.

[18] Manuvakhova M, Keeling K, Bedwell DM (2000). Aminoglycoside antibiotics mediate context-dependent suppression of termination codons in a mammalian translation system.. RNA.

[19] Cassan M, Rousset JP (2001). UAG readthrough in mammalian cells: effect of upstream and downstream stop codon contexts reveal different signals.. BMC Mol Biol.

[20] Howard MT, Anderson CB, Fass U, Khatri S, Gesteland RF (2004). Readthrough of dystrophin stop codon mutations induced by aminoglycosides.. Ann Neurol.

[21] Burke JF, Mogg AE (1985). Suppression of a nonsense mutation in mammalian cells in vivo by the aminoglycoside antibiotics G-418 and paromomycin.. Nucleic Acids Res.

[22] Yang C, Feng J, Song W, Wang J, Tsai B (2007). A mouse model for nonsense mutation bypass therapy shows a dramatic multiday response to geneticin.. Proc Natl Acad Sci U S A.

[23] Morin PJ, Sparks AB, Korinek V, Barker N, Clevers H (1997). Activation of beta-catenin-Tcf signaling in colon cancer by mutations in beta-catenin or APC.. Science.

[24] Fearon K, McClendon V, Bonetti B, Bedwell DM (1994). Premature translation termination mutations are efficiently suppressed in a highly conserved region of yeast Ste6p, a member of the ATP-binding cassette (ABC) transporter family.. J Biol Chem.

[25] Feng YX, Copeland TD, Oroszlan S, Rein A, Levin JG (1990). Identification of amino acids inserted during suppression of UAA and UGA termination codons at the gag-pol junction of Moloney murine leukemia virus.. Proc Natl Acad Sci U S A.

[26] Salas-Marco J, Bedwell DM (2005). Discrimination between defects in elongation fidelity and termination efficiency provides mechanistic insights into translational readthrough.. J Mol Biol.

[27] Kramer EB, Vallabhaneni H, Mayer LM, Farabaugh PJ (2010). A comprehensive analysis of translational missense errors in the yeast Saccharomyces cerevisiae.. RNA.

[28] Lynch SR, Puglisi JD (2001). Structural origins of aminoglycoside specificity for prokaryotic ribosomes.. J Mol Biol.

[29] Schroeder SJ, Blaha G, Moore PB (2007). Negamycin binds to the wall of the nascent chain exit tunnel of the 50S ribosomal subunit.. Antimicrob Agents Chemother.

[30] Yang LY, Trujillo JM (1990). Biological characterization of multidrug-resistant human colon carcinoma sublines induced/selected by two methods.. Cancer Res.

[31] Dexter DL, Spremulli EN, Fligiel Z, Barbosa JA, Vogel R (1981). Heterogeneity of cancer cells from a single human colon carcinoma.. Am J Med.

[32] Bidou L, Stahl G, Hatin I, Namy O, Rousset JP (2000). Nonsense-mediated decay mutants do not affect programmed -1 frameshifting.. RNA.

[33] Oh JH, Ku JL, Yoon KA, Kwon HJ, Kim WH (1999). Establishment and characterization of 12 human colorectal-carcinoma cell lines.. Int J Cancer.

